# Tracheocutaneous Sinus following Tracheocutaneous Fistula Repair: Management Strategies in a Pediatric Patient

**DOI:** 10.1155/2018/6974764

**Published:** 2018-02-18

**Authors:** Adam Bender-Heine, Habib G. Zalzal, Nainika Nanda, Hassan Ramadan

**Affiliations:** ^1^Department of Otolaryngology-Head and Neck Surgery, West Virginia University School of Medicine, Morgantown, WV, USA; ^2^West Virginia University School of Medicine, Morgantown, WV, USA

## Abstract

**Objective:**

To present a rare case of a pediatric tracheocutaneous sinus years after repair of a tracheocutaneous fistula and to review management strategies.

**Background:**

A tracheocutaneous fistula is a common sequela of pediatric tracheostomy and can occur in as many as one in three pediatric patients. There is debate in the literature regarding optimal surgical management.

**Case Presentation:**

An 8-year-old girl presented to the emergency department with swelling and erythema over the anterior neck. Clinical exam and diagnostic imaging revealed an underlying tracheocutaneous sinus.

**Discussion:**

Complications following pediatric tracheostomy are common and range in complexity from stomal granulation to tracheocutaneous fistula. There is some debate regarding the optimal surgical management of children with tracheocutaneous fistula following tracheostomy. This report discusses the management of a pediatric child with an unusual tracheocutaneous sinus and reviews the various surgical techniques which have been described for definitive repair.

## 1. Introduction

Advances in perinatal medicine and healthcare access have increased the number of premature infants requiring the assistance of prolonged ventilation. Pediatric tracheostomies are performed most commonly to address an upper airway obstruction, ventilator dependence, or central hypoventilation [1]. It has been demonstrated that pediatric patients who require cannulation beyond 12 months are predisposed to a tracheocutaneous fistula (TCF) [2–4]. In this paper, we present a rare case of a pediatric tracheocutaneous sinus (TCS) five years after TCF repair. This unusual case report will discuss the patient's background and the events preceding the development of the TCS. In addition, commonly used TCF management strategies will be reviewed as the foundation for the management of a pediatric TCS.

## 2. Case Presentation

**Figure 1:**
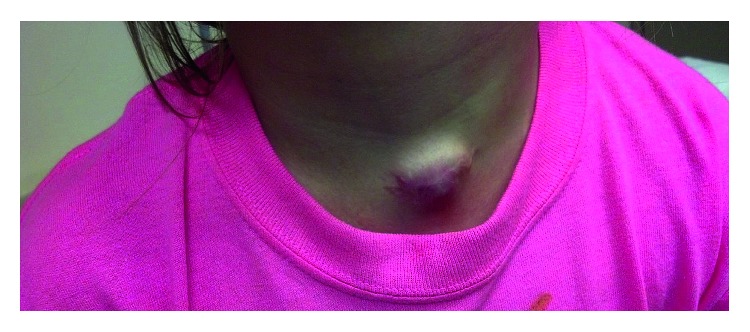
An 8-year-old child with 3 cm diameter anterior midline neck swelling soft and partially compressible on palpation with mild tenderness and intact, mild erythema of overlying skin.

An 8-year-old girl presented to the emergency department with swelling and erythema of the anterior midline neck over a previous tracheostomy scar (Figure 1). The child had been treated with oral antibiotics during the previous 7 days to address mild tenderness and erythema, without swelling, of the anterior neck. Roughly 12 hours prior to the emergency room evaluation, the child's neck acutely ballooned outward over a 3-centimeter diameter without known provocation. The child did not experience dyspnea nor dysphagia at any point.

A brief review of the child's medical and surgical history revealed underlying Stickler syndrome with Pierre Robin sequence. She had undergone a tracheostomy at 2 months of age to bypass an upper airway obstruction. At 2 years of age, the child underwent bronchoscopy and was soon after decannulated. The tracheostomy stoma did not heal spontaneously over 8 months. Therefore, she underwent surgical closure via fistulectomy with four-layered closure a few months before her 3rd birthday. The child did well after surgery. She was seen thereafter only for wellness visits until her emergency room presentation more than 5 years after decannulation at the age of 8 years old.

**Figure 2:**
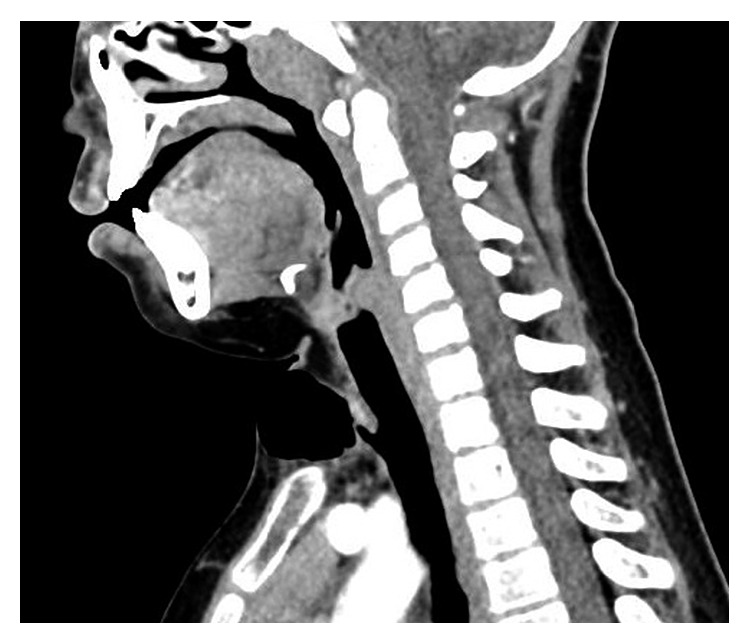
Sagittal plane CT neck demonstrating a small tracheal defect with subcutaneous air trapping to the level of the dermis.

While in the emergency department, the child underwent a computed tomography (CT) scan of her neck, which confirmed the presence of a TCS (Figure 2). The patient was admitted for one evening of observation. After an uneventful night, she was discharged home without needle decompression.

Roughly 2 weeks later, the child returned to the operating room for definitive repair of the TCS, which was accomplished via a modified fistulectomy with four-layered closure and a penrose drain. The small tracheal defect was identified without concomitant bronchoscopy by filling the surgical field with normal saline followed by application of positive pressure ventilation. Once the location of the defect was known, the perichondrium around the tracheal defect was closed with a figure-of-eight absorbable suture, and the overlying soft tissue was closed sequentially in layers. The patient was discharged home the following morning without complication. At subsequent outpatient visits, the neck had healed well.

## 3. Discussion

A tracheocutaneous sinus is a rare sequela following a pediatric tracheostomy relative to TCF, which is a common late complication occurring with a frequency of 6–55% [3, 4]. Subcutaneous emphysema is an early complication after TCF repair and has been reported within the first few weeks following TCF repair [5]. While TCF is common, there are no documented pediatric cases of delayed subcutaneous emphysema or TCS in the literature. Although exceedingly rare, a TCS, or “tracheocele,” is a variant of a TCF and may be addressed in a similar fashion.

Surgical repair of a TCF is important as it directly contributes to poor hygiene, poor phonation, intolerance to submersion, social isolation, and risk of aspiration and infection. TCF can be treated conservatively for the first 3–6 months after decannulation using local curettage, silver nitrate, and cauterization. There is some debate regarding the exact timing of repair; some authors recommend at least a 6-month trial of conservative management to ensure that there is no spontaneous closure [6], whereas other authors may offer a repair after only 3 months [5].

Various techniques for TCF repair have been described, including primary closure without fistulectomy, fistulectomy with healing by secondary intention [7], fistulectomy with four layered closure [4], partial fistulectomy with 3-layered closure [5], and electrocautery-based fistulectomy with healing by secondary intention [6]. At the time of TCF repair, direct laryngoscopy and bronchoscopy are typically performed to assess airway patency [3]. It is important to address any endotracheal fibromas or granulation tissue at the time of TCF repair [5]. After TCF repair, a subsequent airway patency evaluation may be performed at the 1-month postoperative visit [8].

One of the most commonly utilized surgical repair techniques—fistulectomy with multilayered closure—was first described by Joseph et al. [9]. Fistulectomy with multilayered closure involves an elliptical skin excision around the fistula followed by meticulous dissection of the entire fistulous tract to the level of the trachea [9]. A four-layered closure is then performed, including the tracheal defect, strap muscles, subcutaneous fat, and skin. A portion of the strap muscles is often included in the scarred tract, and careful mobilization with realignment of the strap muscles is critical [8]. Alternatively, one may opt to leave a 4 mm stump of fistulous tract which can be closed meticulously with a subsequent three-layered closure [5]. Placement of a rubber band drain between the strap muscles during closure reduces the risk of subcutaneous air tracking, which has been described following TCF repair [5].

In cases where the tracheal defect is large and/or the child has a chronic pulmonary condition associated with frequent and forceful cough, one should consider fistulectomy and healing by secondary intention. In these cases, it is reasonable to discuss standard fistulectomy with healing by secondary intention or fistula tract obliteration via electrocautery since both methods have reported success [6, 10]. A 2017 literature review of TCF repairs had equivalent overall rates of success and complications after fistulectomy followed by primary closure versus closure by secondary intention [10].

A pediatric TCS following tracheostomy is a rare sequela of a pediatric tracheostomy. This case represents an unusual delayed dehiscence of a TCF repair resulting in a pediatric tracheocutaneous sinus. The prolonged cannulation in conjunction with an underlying collagen disorder—Stickler syndrome—may explain this rare TCS [11]. Although there were no specific inciting events identified, we hypothesize that slowly developing tracheal microperforations and a low-grade upper respiratory infection with cough were likely the initiating factors. While a pediatric TCS is exceedingly rare, the diagnosis is relatively straightforward and the management closely parallels that of a TCF.
